# Digital Health Intervention Design and Deployment for Engaging Demographic Groups Likely to Be Affected by the Digital Divide: Protocol for a Systematic Scoping Review

**DOI:** 10.2196/32538

**Published:** 2022-03-18

**Authors:** Catherine L Jenkins, Sumayyah Imran, Aamina Mahmood, Katherine Bradbury, Elizabeth Murray, Fiona Stevenson, Fiona L Hamilton

**Affiliations:** 1 North East London Foundation Trust Essex United Kingdom; 2 eHealth Unit Research Department of Primary Care & Population Health University College London London United Kingdom; 3 School of Psychology University of Southampton Southampton United Kingdom

**Keywords:** digital divide, digital health interventions, DHIs, eHealth, digital health literacy, health inequalities, health inequities, mHealth, mobile health

## Abstract

**Background:**

Digital health interventions refer to interventions designed to support health-related knowledge transfer and are delivered via digital technologies, such as mobile apps. Digital health interventions are a double-edged sword: they have the potential to reduce health inequalities, for example, by making treatments available remotely to rural populations underserved by health care facilities or by helping to overcome language barriers via in-app translation services; however, if not designed and deployed with care, digital health interventions also have the potential to increase health inequalities and exacerbate the effects of the digital divide.

**Objective:**

The aim of this study is to review ways to mitigate the digital divide through digital health intervention design, deployment, and engagement mechanisms sensitive to the needs of digitally excluded populations.

**Methods:**

This protocol outlines the procedure for a systematic scoping review that follows the methodology recommended by the PRISMA-ScR (Preferred Reporting Items for Systematic Reviews and Meta-Analyses Extension for Scoping Reviews) guidance. The following databases will be searched for primary research studies published in English from October 1, 2011, to October 1, 2021: Cochrane Library, Epistemonikos, NICE Evidence, PROSPERO, PubMed (with MEDLINE and Europe PMC), and Trip. In addition, the following sources of gray literature will be searched: Conference Proceedings Citation Index, Health Management Information Consortium, International HTA Database, OpenGrey, The Grey Literature Report, Google Scholar Basic Search UK, MedNar Deep Web Search Engine, and Carrot2. We will select publications that meet the following inclusion criteria: primary research papers that evaluated digital health interventions that describe features of digital health intervention design and deployment that enable or hinder access to and engagement with digital health interventions by adults from demographic groups likely to be affected by the digital divide (eg, older age, minority ethnic groups, lower income, and lower education level). A random selection of 25 publications identified from the search will be double screened by four reviewers. If there is >75% agreement for included/excluded publications, the team will continue to screen all the identified publications. For all included publications, study characteristics will be extracted by one author and checked for agreement by a second author, with any disagreements resolved by consensus among the study team. Consultation digital health intervention design and deployment, and digital health intervention users will also be conducted in parallel.

**Results:**

The review is underway and is anticipated to be completed by September 2022.

**Conclusions:**

The results will have implications for researchers and policy makers using digital health interventions for health improvement peripandemic and post pandemic, and will inform best practices in the design and delivery of digital health interventions.

**International Registered Report Identifier (IRRID):**

PRR1-10.2196/32538

## Introduction

### Background

The COVID-19 pandemic has accelerated widespread adoption of digital health interventions internationally. This rapid shift to digital delivery has laid bare the impact of pre-existing and emerging systemic health inequalities on communities most in need of accessible health care, specifically people from ethnic minority backgrounds, people from lower socioeconomic backgrounds, older people (aged ≥65), and people living with disabilities [1,2]. Belonging to one or more of these groups (intersectionality) is a risk factor for experiencing more severe illness and mortality [3]. These same groups are likely to make up a large proportion of people vulnerable to health inequalities regardless of initial illness severity [4].

Health inequalities have been defined as “the systematic, avoidable and unfair differences in health outcomes that can be observed between populations, between social groups within the same population or as a gradient across a population ranked by social position” [5], but the term is also used for differences in access to health care, quality of care received, wider determinants of health such as housing and education, and opportunities to lead healthy lives, including differences in risky behaviors such as smoking [6]. The related term “health inequity” implies a normative judgement about the fairness or otherwise of these differences, “expressing a moral commitment to social justice,” as described by Kawachi et al [7]. For this review, we use the term health inequalities.

The digital divide—the gap between populations able to benefit from access to and use of health information and services online and populations unable to take up such opportunities—is a clear example of health inequality and exacerbates the inverse care law [8] such that digitally delivered health care runs the risk of excluding the people who could most benefit. This review seeks to address issues of social justice in the digital delivery of health care by providing a comprehensive overview of the literature on strategies to reduce the digital divide through the design and deployment aspects of digital health interventions.

### The Digital Divide and Digital Health Literacy

Health literacy and digital literacy are both key determinants of health [9]. The term digital health literacy (also referred to as eHealth literacy) brings both literacies together to describe the degree to which individuals can access, understand, and apply digitally delivered health information and services to make informed decisions about their health. Importantly, digital health literacy extends beyond personal responsibility to encompass the responsibility of digital health systems and services to support and dynamically respond to the digital health literacy skills, and the confidence and motivation to develop such skills, in the populations they serve [10].

Digital health literacy competence fluctuates depending on context, but a clear link has been demonstrated between low digital health literacy and poor health outcomes [11]. The accelerated shift to digital health care to comply with social distancing measures arising from COVID-19 heightens the risk of poor health outcomes in digitally excluded groups and further complicates an already complex interplay between intersectionality and digital health literacy as a distributed, or *outsourced*, concept. For example, many older people may have the financial capital to afford digitally enabled devices and data plans but not the skills, motivation, or confidence to engage directly with such technology; instead, they may be reliant on their networks—family members, friends—to navigate digital health care on their behalf [12,13].

Digital health intervention design and deployment therefore need to be proportionate and prioritize those groups most affected by low digital health literacy on both individual and systemic levels via a two-pronged approach: first, to develop patients’ skills in accessing, understanding, and using digital health information and services, and second, to develop the digital health literacy responsiveness of the systems and health care professionals (HCPs) supporting digital health intervention deployment in practice. This review will have wider implications for research into digital health interventions and will address:

- The digital divide: How should digital health interventions be designed and deployed to mitigate the digital divide (eg, patient access to WiFi-enabled digital devices or subsidized data plans, the inclusiveness of recruitment methods for studies evaluating digital health interventions, strategies to increase uptake and use of a digital health intervention app by people from demographic groups likely to be affected by the digital divide, or the content of onboarding scripts used by HCPs when introducing patients to a digitally delivered health service)?
- Digital health literacy: How can app design and digital skills training be optimized to reduce digital health inequalities arising from low digital health literacy (eg, integration of user testing into the design process, readability of app content, gamification/social features to engage users, or support for HCPs to train as “digital health champions” as part of improving their own skills)?

### Rationale for Conducting a Systematic Scoping Review

The decision to conduct a systematic scoping review rather than a systematic review is informed by the purpose of scoping reviews, which is to provide a comprehensive picture of the available evidence to guide further research. This purpose is of relevance to the study of digital health interventions because digital health intervention design and deployment is frequently captured in gray literature (eg, charity reports).

This review will be conducted using methods outlined by the Joanna Briggs Institute (JBI) Reviewers’ Manual [14] and will conform to the PRISMA-ScR (Preferred Reporting Items for Systematic Reviews and Meta-Analyses Extension for Scoping Reviews) [15]. The protocol for this review is based on the JBI framework by Peters et al [16] with further process updates from Levac et al [17] and is structured as follows: identification of the review aims and search questions, selection of evidence sources, charting of data extracted from the evidence sources, reporting of results, and consultation with stakeholders (including HCPs, digital health experts, and digital health intervention users). Results from this review will support decision-making when designing and deploying digital health interventions.

### Review Question

The purpose of this systematic scoping review is to identify research that reports on the design and deployment of digital health interventions to reduce the digital divide and increase digital health literacy at macro- (national policy), meso- (national program), and micro- (localized or individual) levels [18]. It aims to do this by discovering peer-reviewed qualitative, quantitative, and mixed methods research, and gray literature relevant to mapping the contextual and process factors that enable or hinder engagement with digital health services by people vulnerable to the digital divide and individual or systemic low digital health literacy.

This review will seek to answer the review question, “What features of digital health intervention design and deployment enable or hinder engagement with digital health interventions by people from demographic groups likely to be affected by the digital divide?”

Health interventions are increasingly delivered through digital platforms, and it is important that they do not exacerbate or create health inequalities. Our hypothesis is that existing knowledge in the literature can inform our objective to find ways to bridge the digital divide and improve digital health literacy in underserved groups, for example, through digital health intervention design, development, and deployment.

**Figure 1:**
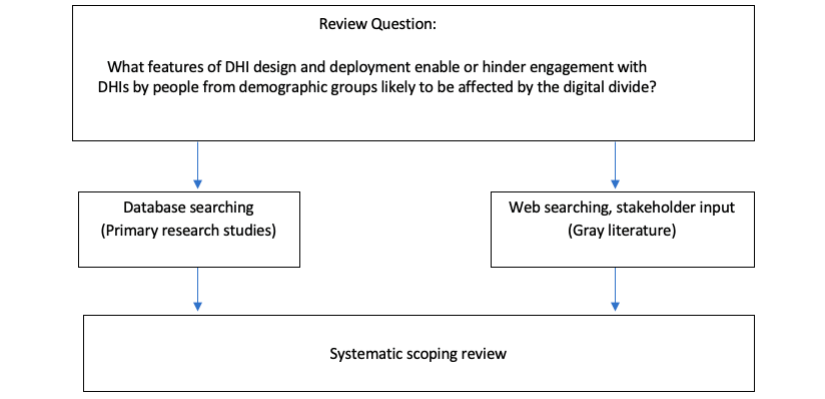
Systematic scoping review flow diagram. DHI: digital health intervention.

Figure 1 outlines how this review will be conducted.

## Methods

We will follow the methods recommended by the PRISMA-ScR framework [15].

### Protocol Registration

At the time of writing, scoping reviews are ineligible for registration in PROSPERO. For transparency and to enable the systematic scoping review method outlined here to be repurposed for onward research, a summary of the protocol and any supplementary material will be registered with the Open Science Framework [19] and assigned a Digital Object Identifier (DOI) for long-term retrieval.

### Eligibility Criteria

We will include primary research studies reporting on the design or deployment of digital health interventions that meet the following inclusion criteria.

#### Population

Adults from different demographic groups (defined based on gender, age, ethnicity, income, and education) in high-income and low-income countries.

#### Intervention

Digital health interventions are defined as any service intended to improve physical or mental health, or to promote health improvement through, for example, lifestyle change delivered digitally (formally or informally), such as via smartphone apps, social media, email, SMS text message, using wearable technologies, video games (eg, for motor or cognitive training), websites, or telehealth (eg, remote consultations) but excluding telemedicine if this consists solely of remote monitoring without any input from the patient [15].

#### Comparator

There may or may not be a comparator, depending on the study design.

#### Outcome

The primary outcome of interest is the capacity to mitigate the digital divide and increase digital health literacy either through the digital health intervention itself or its deployment. Health impact is not considered an outcome of interest for this review.

- “Mitigate the digital divide” refers to removing barriers to digital inclusion, for example, an intervention that provides free smartphones or tablets, or low-cost cellular data or WiFi for people with low incomes [10].
- “Increase digital health literacy” refers to digital skills development [9], either through the design of the digital health intervention or through programs to deploy the digital health intervention, resulting in higher levels or use or confidence in using the digital health interventions, measured either by interview or self-report (eg, the eHealth Literacy Scale questionnaire [20] or use data). Changes in these outcome measures suggest that both users and the health professionals administering the digital health intervention are confident in the use of digital resources for safe and sustainable self-management and to promote safe and sustainable self-management.

For the purposes of this review, gray literature covers conference abstracts and proceedings, white papers, and stakeholder reports (eg, by charities with an interest in digital literacy or who promote the health and well-being of people from different demographic groups) that enhance the contextual understanding of the field.

There will be no limitation by geography because lessons from low-income countries could inform strategies to reduce digital health inequities in high-income countries. We will report and interpret data within the context of the country where the study was based.

Limitations will be placed on user population and on study date. The user population will prioritize the marginalized demographics previously outlined; the study date will be limited to post-2011 (for preprint or publication) in recognition of the rapid change of pace in digital health intervention development [21]. No limitations will be placed on the study design or publication status. However, we will limit included studies to those published in English.

Decisions on whether data should be included or excluded from further analysis will be guided by criteria that, in line with a systematic application of scoping review method [16], will be iteratively refined based on increasing familiarity with the literature under review.

The use of the term digital health intervention is a possible challenge as it covers a range of digital health technologies [22] and may contribute to an imbalance in this review that favors comprehensiveness over precision. This will be addressed by working closely with information specialists to peer review the preliminary search strategy in line with the PRESS (Peer Review of Electronic Search Strategies) procedure [23], ensuring a feasible and focused approach that is also flexible enough to be iteratively reworked in response to the results retrieved.

### Evidence Sources

#### Reviews

A preliminary search for registered, preprint, or published systematic, scoping, and other review types will be conducted to pilot the search strategy, covering the following databases (via the Ovid interface, where applicable):

- Cochrane Library
- Epistemonikos
- NICE Evidence
- PROSPERO
- PubMed (with MEDLINE and Europe PMC)
- Trip

The preliminary search will provide keywords that can be incorporated into searches across evidence tiers below that of the gold standard represented by reviews. It will also serve to identify Medical Subject Headings (MeSH) and PubMed IDs, which can be inserted into the Yale MeSH Analyzer [24] to extract further MeSH and to identify keywords to capture preprints not yet indexed with MeSH. A cap of 100 review articles will be applied in cases where unfeasible amounts of hits are retrieved.

Model papers for citation “*pearl-growing*”—papers already identified as highly relevant to this review—will be mined for their keywords and applied as test cases for honing the search strategy. Vernacular search terms will be identified from hand searching, including reference list scanning, forward/backward citation snowballing, and table of contents scanning of relevant journals online, such as the *Journal of Medical Internet Research* and its sister journals.

Further sources for other tiers of evidence include:

- Databases of peer-reviewed primary research (qualitative, quantitative, mixed methods studies):
  CINAHL
  EMBASE
  PsycINFO
  OTseeker
  PubMed
- Gray literature
  Conference Proceedings Citation Index
  Health Management Information Consortium
  International HTA Database
  OpenGrey
  GreyNet
  The Grey Literature Report

The Grey Literature Report [25], available up to 2016, is a key source of gray literature that, unusually, is indexed using MeSH. It may be helpful in providing alternative MeSH to feed back into the search strand for peer-reviewed literature.

Alongside database searching, web searching will be conducted systematically, to the extent that this is feasible given that web searching is vulnerable to changing web content and algorithms [26]. Search terms will be used consistently between database and web searching and screen captures of content, and where available, DOIs will be saved for transparency. Web searching will also support the discovery of human-computer interaction (HCI) literature, which is not reliably indexed in MEDLINE. Searching the HCI literature will be helpful for the provision of further perspectives on design challenges (eg, designing for people with low vision or motor impairments).

#### Web Searching

**Figure 2:**
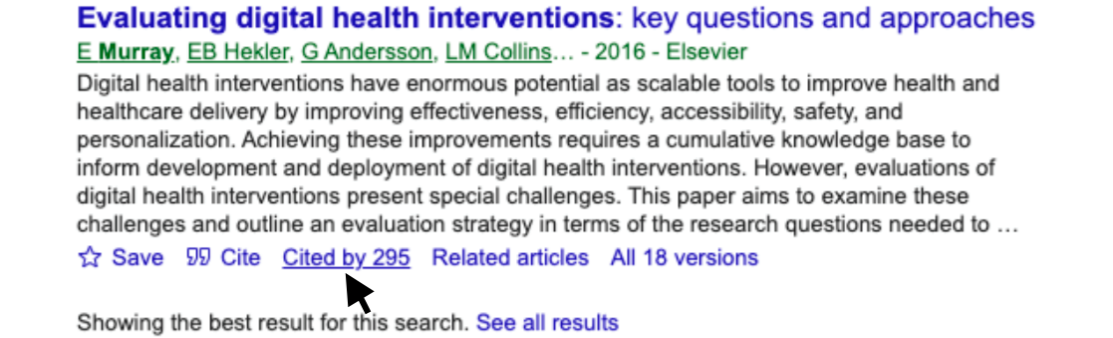
"Cited by" feature for forward citation snowballing in Google Scholar.

The following databases will be used for the web searching: Google Scholar Basic Search UK, with use of the “Cited by” function (see Figure 2), for the first 100 results retrieved; MedNar Deep Web Search Engine, for the first 100 results retrieved; and Carrot2.

Targeted consultation with experts in digital health intervention design and deployment and digital health intervention users will also be conducted in parallel. This is part of the added value of scoping reviews. Ethics approval is not required for such consultation.

### Search Strategy

The following search strategy was used for PubMed (November 2021): ((“digital health” OR “digital health intervention*” OR “DHI” OR “digital rehabilitation”) OR “eHealth” OR “mHealth” AND (app OR intervention OR technology) AND (barrier* OR design OR disparit* OR divi* OR engage* OR exclu* OR inequ*)) AND literacy.

This search strategy will be modified by incorporating MeSH and keywords used to index relevant papers and will inform the search strings for parallel searching in Google Scholar Advanced Search UK and MedNar.

Screening of identified literature will follow the recommendations of the JBI [16]. Of the studies and gray literature initially identified from the searches, 25 publications will be selected at random by dividing the total by four (the number of screeners), then selecting every nth paper (where n=the total divided by four) to assign for double-screening of titles, abstracts, or full-text by four reviewers; if there is greater than 75% agreement for included/excluded publications, then the team will continue to screen all of the identified publications. If agreement is 75% or less, we will hold a further training session to resolve differences in interpretation of the criteria and, if necessary, tighten the wording. Further rounds of double-screening and calculation of interrater agreement will continue until we meet concordance. For included publications, study characteristics will be extracted by one author and checked for agreement by a second author, and any uncertainty over data retrieved will be discussed and resolved via consensus with all contributing authors. Retrieval of a model paper [27] served as the test case for the search strategy.

### PRISMA-ScR

The PRISMA-ScR framework will be used to transparently record the selection, deduplication, and screening decision process, supplemented by updated recommendations from Tricco et al [28]. Gray literature may not be searchable by abstract (due to a lack of abstract fields in the gray literature discovery interfaces), so the PRISMA-ScR flow diagram will be adapted to accommodate this. Critical appraisal, for example, of the underlying evidence informing the studies retrieved, is not generally operationalized in scoping reviews [16]. Instead, the focus of this review type is on providing as complete a picture as possible of what is currently known about the topic of interest.

### Data Extraction and Charting

Data will be extracted from full-text sources relevant to the review question using the JBI data extraction guidance for scoping reviews [14]. If required, missing data will be requested from authors. Google Sheets [29] will be used to record the initial data extraction of all included sources. In accordance with the iterative approach taken by scoping reviews, the following headers will be trialed and refined as needed [30]:

- Reference
- Region and setting of digital health intervention design/deployment
- Study type (qualitative, quantitative, mixed methods, review)
- Funding source
- Stakeholder involvement
- Digital health intervention type
- Digital health intervention purpose
- User population
- Summary of results relevant to this review question

### Collating, Summarizing, and Reporting Results

Unlike a systematic review—where evidence sources that do not meet the quality criteria applied are excluded—scoping reviews seek to present an overview of all material reviewed (including gray literature) [24]. We will summarize the results visually [30] to assist validation of the results by stakeholders (described in the following section) and later dissemination.

### Consultation Exercise

In parallel with the aforementioned stages, consultation with stakeholders (eg, representatives from charities, experts in researching digital health interventions, and patients or members of the public) will be conducted to validate emerging results. Stakeholders will be identified both a priori (known to the authors) and iteratively as this review progresses (through their representation in relevant sources).

## Results

This systematic scoping review is in progress. The final draft of this review will be submitted by September 2022. We expect to report a narrative summary of the findings of included peer-reviewed primary studies and gray literature that describe differences in the ability of people from different demographic groups or people with lower health literacy or digital health literacy to access and use digital health interventions, and any change in this following specific measures taken by the researchers to make digital health interventions more accessible and usable by people from these groups; and the results of qualitative studies that discuss barriers and facilitators to access and use digital health interventions by people from different demographic groups.

## Discussion

This review will present a summary of evidence regarding strategies for optimizing the design and deployment of digital health interventions to mitigate the effects of the digital divide and low digital health literacy on populations disproportionately affected by health inequalities and the digital divide. Its results will inform the design and deployment of digital health interventions at a time when they are more used than ever before and will have wider implications for researchers and policy makers using digital health interventions for health improvement. For example, HCI strategies for developing content for websites and apps, such as use of personas with a range of demographic characteristics (including those of digitally excluded populations) [31,32] and use of video and audio to help people of low health literacy use digital health interventions [33].

In conclusion, this review is among the first to examine the design and deployment of digital health interventions specifically in the context of the challenges presented by the digital divide and low digital health literacy at individual and systemic levels. The results of this review will support researchers, digital health intervention developers, and HCPs in identifying what works to optimize digital health intervention design and deployment, with the aim of promoting social justice.

## Abbreviations

DOI: Digital Object Identifier
HCI: human-computer interaction
HCP: health care professional
JBI: Joanna Briggs Institute
MeSH: Medical Subject Headings
NIHR: National Institute for Health Research
PRESS: Peer Review of Electronic Search Strategies
PRISMA-ScR: Preferred Reporting Items for Systematic Reviews and Meta-Analyses Extension for Scoping Reviews
